# Neonates and Young Infants With COVID-19 Presented With Sepsis-Like Syndrome: A Retrospective Case Controlled Study

**DOI:** 10.3389/fped.2021.634844

**Published:** 2021-02-25

**Authors:** Manasik Hassan, Ahmed Khalil, Samar Magboul, Ohood Alomari, Tasneim Abdalla, Hafez Alsliman, Abdulla Alhothi, Eman Al Maslamani, Mohammed AlAmri, Ashraf Soliman

**Affiliations:** ^1^Section of Academic General Paediatrics, Department of Paediatrics, Hamad General Hospital, Doha, Qatar; ^2^Department of Clinical Pharmacy of Paediatric, Hamad General Hospital, Doha, Qatar; ^3^Section of Paediatric Emergency, Department of Paediatrics, Hamad General Hospital, Doha, Qatar; ^4^Department of Paediatrics, Hamad Medical Corporation, Doha, Qatar

**Keywords:** neonates, young infants, COVID-19, fever, sepsis

## Abstract

**Objective:** We aimed to describe the presentations and biochemical characteristics of sepsis-like syndrome (SLS) in infants aged <2 months who tested positive for SARS-CoV-2-in comparison to those in the same age group who were SARS-CoV-2-negative.

**Background:** COVID-19 presents with a spectrum of manifestations, and children seem to have a favorable clinical course compared to other age groups. Limited data are available for symptomatic infants.

**Design:** This was a case-controlled single-institution retrospective study on infants aged <2 months admitted with SLS between 1 April 2020 and 1 July 2020. These infants were divided into 2 groups: Group 1 (*n* = 41), infants with positive nasal/oropharyngeal swab polymerase chain reaction (PCR) results for SARS-CoV-2; and Group 2 (*n* = 40), infants with negative PCR results for SARS-CoV-2 (control group). Details between both groups were reviewed and analyzed.

**Outcome:** The clinical and laboratory data for SARS-CoV-2 -positive infants who presented with SLS may differ from those for infants with SLS who tested negative for SARS-CoV-2.

**Results:** Overall, 105 infants were admitted with clinical sepsis: 41 were SARS-CoV-2-positive, and 64 were negative. Fever was present in 90% of SARS-CoV-2-positive infants vs. 80% of the negative group. SARS-CoV-2-positive infants had a higher incidence of nasal congestion and cough (39 and 29%, respectively) compared to the SARS-CoV-2-negative group (20 and 3%, respectively) (*P* < 0.05). Poor feeding and hypoactivity occurred more frequently in the SARS-CoV-2-negative group (58 and 45%, respectively) than in the SARS-CoV-2-positive group (22 and 12%, respectively, *P* < 0.004). Sepsis workup, including lumbar puncture, was performed in 67% and partial septic workup was performed in 23% of the SARS-CoV-2-positive infants. Full sepsis workup was performed in 92% of the SARS-CoV-2-negative group. Cerebrospinal fluid (CSF) cultures were negative in 26/27SARS-CoV-2-positive infants (an infant had *Klebsiella meningitis*). All the SARS-CoV-2-negative infants had negative CSF cultures. Blood culture was negative in both groups. Urine culture showed bacterial growth in 9 infants with SARS-CoV-2-negative sepsis.

**Conclusions:** Our study showed that respiratory symptoms (cough and nasal congestion) were more prominent in the SARS-CoV-2-positive group, while poor feeding and hypoactivity were reported more frequently in the negative group. However, the clinical differentiation between COVID-19 disease and sepsis in such age groups is difficult. Therefore, screening young infants with SLS for SARS-CoV-2- is necessary during this pandemic.

## Introduction

The transmission of coronavirus disease-2019 (COVID-19) to young infants and neonates is thought to occur fundamentally through respiratory droplets during the post-natal period when they come in contact with their SARS-CoV-2-positive mothers. Knowledge about the magnitude, clinical course, and outcome of COVID-19 infection in this young age group is limited (1).

The described clinical manifestations among neonates with SARS-CoV-2 virus included fever, lethargy, rhinorrhea, cough, tachypnea, vomiting, diarrhea, rash, and poor feeding. However, the degree to which SARS-CoV-2 has contributed to these manifestations is uncertain. Many of these clinical findings can be due to other causes (e.g., bacterial sepsis, metabolic derangement, transient tachypnea of the new-borns, and neonatal respiratory distress syndrome) (2).

Recent evidence suggests that COVID-19 infection is uncommon in neonates. Most SARS-CoV-2-positive infants had either asymptomatic infections or mild disease with excellent recovery. Severe clinical courses of COVID-19 infection in neonates, which required assisted and/or mechanical ventilation, have been reported. A large study of pediatric SARS-CoV-2 cases (suspected or confirmed) in China between the end of December 2019 and February 2020 reported that 11% of infants had a severe or critical illness (3–6). In addition, many sporadic cases of COVID-19 infection have been reported with a wide range of severity (7, 8).

Zeng et al. (9) described a group of 33 infants from mothers with SARS-CoV-2, and three of them were symptomatic with radiologically proven pneumonia. These three infants had a severe clinical course but had favorable outcomes. Polymerase chain reaction (PCR) results for SARS-CoV-2-were negative 6–7 days after the onset of symptoms. None of these new-born infants died (10).

Pre-term neonates and those with other underlying medical conditions appeared to have a higher risk of developing severe disease. They presented with insidious and non-specific symptoms such as lethargy and even dehydration (11–13). A pre-term neonate developed sepsis caused by Enterobacter species and coagulopathy, which resolved with the recovery of sepsis (10).

The limited data and variable clinical course of the disease in symptomatic new-borns and young infants with COVID-19 infection mandate detailed research and analysis to comprehend the disease within this age group.

In this study, we evaluated the clinical and biomedical characteristics of a cohort of neonates and young infants who presented with symptoms and signs suggestive of sepsis-like syndrome [SLS] and were found to be infected with severe acute respiratory syndrome coronavirus 2 (SARS-CoV-2). Their data were compared with a group of new-borns who were admitted with SLS and proved to be SARS-CoV-2-negative. In addition, we assessed the associated comorbidities and outcomes of these infants.

## Materials and Methods

### Study Design

This case controlled, single-institution, retrospective observational study was conducted in Hamad General Hospital (HGH), The State of Qatar. It included infants of <8 weeks of age who were admitted with SLS during the COVID-19 pandemic (between 1/4/2020 and 1/7/2020). These infants were divided into two groups according to nasal and oropharyngeal swab PCR testing for SARS-CoV-2; all infants with positive oropharyngeal swab PCR and a control group (negative swab PCR) in which random tables were used to randomize the control group. Clinical and laboratory data of SARS-CoV-2-positive infants were compared to those of SARS-CoV-2-negative infants (control). Details of the presenting symptoms and signs, biochemical lab profile, duration of hospital stay, antibiotic use, and patient outcome were reviewed and analyzed in both groups.

### Inclusion and Exclusion Criteria

This is a chart-based review study for all neonates and infants aged <8 weeks at diagnosis who were admitted to the hospital with SLS, including one or more of these manifestations: fever, hypotonia, poor feeding, diminished spontaneous activity, less vigorous sucking, apnea, bradycardia, temperature instability, respiratory distress, vomiting, diarrhea, abdominal distention, jitteriness, seizures, and/or jaundice. Exclusion criteria included all pediatric cases above 8 weeks of age.

### Data Collection

All clinical, laboratory, and radiological data were collected retrospectively by reviewing the electronic hospital medical records (Cerner) of the patients for all infants.

### Data Analysis

Descriptive comparative statistics were used to summarize all characteristics of the participants. Student's *t*-test was used to compare variables between the two studied groups when data were normally distributed, and the Mann–Whitney *U*-test was used when the data were not normally distributed. A two-sided *P* < 0.05 was considered significant.

### Ethical Approval

The study was conducted in conformance with the principles of the “Declaration of Helsinki,” Good Clinical Practice, and within the laws and regulations of MOPH in Qatar after the approval of the institutional review board committee in HGH, Doha, Qatar with approval number MRC-01-20-566.

## Results

Overall, 105 infants aged <8 weeks were admitted to the pediatric ward of HGH with SLS between 1 April and 1 July 2020. Forty-one infants were SARS-CoV-2-positive and sixty-four were negative (40 of them were randomly selected to serve as controls). The male: female ratio was 0.8:1 in both groups. The SARS-CoV-2-positive group was significantly older than the negative group (mean age 32 vs. 17 days, respectively) (*P* < 0.001) at the time of presentation. Gestational age did not differ between the two groups. Eight infants were premature (three were SARS-CoV-2 positive and five were SARS-CoV-2-negative) (Table 1).

**Table 1:** Demographic data for both groups.

	**COVID-19 positive neonates *N* = 41**	**COVID-19 negative neonates *N* = 40**	***P*-value**
Gestational age (weeks)	38.3 (36–40)	38.5 (34–40)	0.4
Prematurity (<37 weeks)	3 (7%)	5 (7.8%)	0.8
Age at presentation (days)	32 (8–49)	17 (2–49)	0.001*
Gender: Male	19 (46%)	17 (43%)	
Female	22 (54%)	23 (57%)	0.7
Male to female ratio	0.9:1	0.7:1	
Mode of delivery:			
Normal vaginal delivery	29 (71%)	28 (70%)	0.9
C-section	12 (29%)	12 (30%)	

* *P < 0.05*.

Fever was present in 90% of SARS-CoV-2-positive infants vs. 80% of the negative group. SARS-CoV-2-positive infants had a higher occurrence of nasal congestion and cough (39 and 29%, respectively) than the negative group (20 and 3%, respectively) (*P* < 0.05). SARS-CoV-2-positive cases had more instances of diarrhea and vomiting (15 and 7%, respectively) than the SARS-CoV-2-negative infants (8 and 5%, respectively). Poor feeding and hypoactivity were significantly higher in the SARS-CoV-2-negative group (58 and 45%, respectively) than in the positive group (22 and 12%, respectively) (*P* < 0.004) (Table 2).

**Table 2:** Clinical characteristic for both groups.

**Symptoms and signs *N* (%)**	**COVID-19 positive neonates *N* = 41**	**COVID-19 negative neonates *N* = 40**	***P*-value**
Fever	36 (90%)	32 (80%)	0.2
Nasal congestion	16 (39%)	8 (20%)	0.03*
Cough	12 (29%)	1 (3%)	0.009*
Poor feeding	9 (22%)	23 (58%)	0.004*
Hypo-activity	5 (12%)	18 (45%)	0.004*
Irritability	5 (12%)	8 (20%)	0.17
Diarrhea	6 (15%)	3 (8%)	0.15
Vomiting	3 (7%)	2 (5%)	0.33
Tachypnea	0 (0%)	1 (3%)	0.16
**Vital sings (Mean)**			
Temperature axillary (36.5–37.5)	38.10	38.25	0.12
Heart rate (normal range 110–160)	165.39	158.45	0.07
Respiratory rate (30–60)	45.10	44.13	0.22
Blood pressure systolic (65–85)	87.83	82.10	0.001*
Blood pressure diastolic (45–55)	51.51	45.90	0.001*
Oxygen saturation (>94%)	41 (100)%	40 (100)%	

* *P < 0.05*.

Total white cell counts were significantly lower in the SARS-CoV-2-positive group than in the negative group (mean 8.42 × 10^9^/L vs. 12.5 × 10^9^/L, respectively) (*P* < 0.001). The absolute neutrophil count (ANC) was lower in the SARS-CoV-2-positive group than in the negative group (mean 2 × 10^9^/L vs. 5 × 10^9^/L, respectively) (*P* = 0.001). Lymphocyte percentage was higher in the SARS-CoV-2-positive group than in the negative group (59 vs. 41%, respectively). Serum albumin was lower in the SARS-CoV-2-positive group than in the negative group (mean 35 vs. 44 g/L, respectively) (*P* < 0.001). C-reactive protein (CRP) was significantly elevated in SARS-CoV-2-negative vs. SARS-CoV-2-positive infants (42 vs. 5%, respectively) (*P* 0.005) (Table 3).

**Table 3:** Biochemical characteristics for both groups.

	**COVID-19 positive neonates *N* = 41 Mean (average)**	**COVID-19 negative neonates *N* = 40 Mean (average)**	***P*-value**
White blood cells (×10^9^/L) Reference range (10.8– 18.1)	8.42 (3.9–12.8)	12.22 (5.7–24.1)	0.001*
Hemoglobin (g/dL) Reference range (13.9–16.5)	12.47 (9.2–18.2)	15.43 (9.7–21.8)	0.001*
ANC counts (×10^9^/L) Reference range (3.8–11)	2 (0.6–4.7)	5.38 (0.3–15.7)	0.001*
ANC % Reference range (35–61)	22 (7–57)	40 (18–68%)	0.001*
Lymphocyte counts (×10^9^/L) Reference range (5.5–6)	4.72 (1.5–9.1)	4.9 (1.3–13.4)	0.6
Lymphocyte % Reference range (31–56)	59 (19–84)	41 (15–78)	0.001*
Platelet counts (×10^9^/L) Reference range (192–252)	369 (223–666)	384.48 (187–634)	0.6
CRP >5 (cases no.) Reference range (<5)	2 (5%)	17 (43%)	0.005*
Serum albumin (g/L) Reference range (35–44)	35 (28–41)	44 (32–44)	0.001*
CSF study analysis	20 (50%)	28 (70%)	
RBC (cell/ul) Reference range (0–2)	55 (0– 500)	80 (0–759)	0.13
WBC (cell/ul) Reference range 0–30)	19 (2–307)	11 (2–113)	0.11
Glucose CSF mmol/l	2.7 (2.1–3.8)	2.6(1.9–3.9)	
Reference range (2.4–4)			0.40
Glucose in serum	4.9 (4.2–5.5)	4.49 (2–6.9)	
Glucose CSF/ serum	55%	62%	
Abnormal value >60%			
CSF protein g/l Reference range (<0.4)	0.69 (0.34–3)	0.76 (0.58–2.14)	0.02*
CSF culture	(3%)	0 (0%)	–
CSF viral meningitis	0 (0%)	0 (0%)	–
Urine culture	Negative in 41 (100%)	Positive in 9 (23%) Negative in 31 (77%)	
Stool culture	Negative in 4/4 (100%)	Negative in 4/4 (100%)	
Respiratory co-infection	–	Rhino virus 4/18 (22%)	
CT value Non-infectious >30	17 (14–28)	–	

* *P < 0.05*.

There was no significant difference between the total cerebrospinal fluid (CSF)-white blood cell (WBC) count, total CSF-red blood cell count, CSF glucose/serum ratio, and CSF protein between the SARS-CoV-2-positive vs. the SARS-CoV-2-negative group (Table 3).

CSF cultures were negative in 26/27 patients of the SARS-CoV-2-positive group (one case had *Klebsiella meningitis*). The entire SARS-CoV-2-negative group had negative CSF cultures. Both groups had negative blood cultures. Nine patients of the SARS-CoV-2-negative group had positive urine cultures in which six cases were *E.coli*, two were *Klebsiella*, and only one case was *Enterococcus faecalis* while none of the SARS-CoV-2-positive infants had positive urine cultures.

Four patients of the SARS-CoV-2-negative group showed Rhinovirus in their respiratory viral screening. The mean cycle threshold (CT) value for the SARS-CoV-2-positive group was 17, denoting a substantial load (Table 3).

Plain chest radiographs were obtained for 36/41 patients in the SARS-CoV-2-positive group. Radiological changes were mostly mild and non-specific. No chest changes with clear lungs and normal pulmonary vascularity occurred in 6.2% of infants. Slightly accentuated central lung vascularity occurred in 12.5% of patients. Prominent hilar shadows with increased linear lung shadowing, mainly peri-hilar and pre-cordial, were detected in 34% of patients. Increased linear lung shadowing with a tiny bead-like appearance, more peripherally distributed, and at the lower lobes, occurred in 28% of patients. Superadded peri-bronchial cuffing, perivascular haziness, and lace-like fine linear appearance of the interstitium occurred in 9.3% of patients. Superadded ground-glass fine opacities (GGO) scattered in two or more lobes of both lungs occurred in 15.6% of patients. Only 6.2% of patients showed superadded consolidations in both lungs, mainly peripherally oriented (Table 4).

**Table 4:** Chest radiographic findings in COVID-19 positive infants.

**Radiological changes**	**No. of patients**	**%**
Clear both lungs and normal pulmonary	2	6.2%
Accentuated central lung vascularity	4	12.5%
Prominent hilar shadows +increased linear shadows	11	34.3%
The above + tiny, beaded appearance	9	28.1%
Above + peri-bronchial cuffing, perivascular haze, lacelike fine lung reticulations	3	9.3%
Above + ground glass opacities	5	15.6%
Above + consolidation	2	6.2%
Hilar enlargement	1	3.1%

SARS-CoV-2-negative neonates required a longer hospital stay compared to the SARS-CoV-2-positive group (8 vs. 3.7 days) (*P* < 0.001). Admission to the pediatric intensive care unit (PICU) was required more often in the SARS-CoV-2-negative (18%) than in the SARS-CoV-2-positive (3%) infants. The length of PICU stay did not differ between the two groups. Antibiotics had been started empirically according to our local policy for the management of neonatal sepsis (Cefotaxime and Ampicillin) in which the duration of treatment was significantly shorter in the SARS-CoV-2-positive group (3.7 days) than in the SARS-CoV-2-negative group (7.5 days; *P* < 0.03). All infants in both groups recovered fully with no comorbidities (Table 5).

**Table 5:** Hospital stay and antibiotic administration.

	**COVID positive neonates *N* = 41**	**COVID negative neonates *N* = 40**	***P*-value**
PICU admission N (%)	1 (3%)	6 (18%)	0.06
PICU length of stay (days)	6.5	6.3	0.09
Anti-biotic administration N (%)	36 (90%)	40 (100%)	0.02
Anti-biotic duration (days)	3.7	7.5	0.03
Length of hospital stay (days)	4.5	8	0.001
Recovered fully	41 (100%)	40 (100%)	0.4

## Discussion

Specific data concerning the clinical course of COVID-19 in neonates and young infants aged <2 months are limited. The reported case series either combined the results of all infants <1 year of age or completely excluded them (1, 2). We studied 81 neonates and young infants who presented with sepsis-like manifestations. Forty-one patients were SARS-CoV-2-positive. All patients had a mild illness with good outcomes and no mortality (4). The course of SARS-CoV-2 in this age group appeared to be similar to that of other viral respiratory infections (5, 6, 14).

Overall, 90% of our infants with COVID-19 infection had a fever, which appeared more frequently compared to children reported from China (44–85%) (2, 7, 11). In addition to fever, the main symptoms of COVID-19 infection in new-borns are respiratory (cough and nasal congestion) and gastrointestinal. On the other hand, irritability, poor feeding, and hypoactivity were the chief presentations of SARS-CoV-2-negative babies. Unlike the higher prevalence of COVID-19 infection in males reported in many studies, females accounted for 54% of cases in our study (15–17).

In the course of COVID-19 infection, indications of inflammation might include elevated levels of CRP, erythrocyte sedimentation rate, fibrinogen, pro-calcitonin, D-dimer, ferritin, lactic acid dehydrogenase or interleukin 6, and neutrophils and reduced lymphocytes and low albumin levels (18).

Our new-borns with COVID-19 infection had significantly lower WBC and ANC with relative lymphocytosis compared to the SARS-CoV-2-negative infants. A previous report on children with COVID-19 infection reported decreased WBC count with marked lymphopenia and neutrophilia (19–21).

The finding of normal CRP and low WBC, and ANC in our infants with COVID-19 infection complemented the mild inflammatory reaction and the relatively mild clinical course of the disease in this age group.

Lower albumin levels were observed in our SARS-CoV-2-positive infants than in the SARS-CoV-2-negative infants, despite other normal hepatic functions (ALT, AST, and bilirubin). In support of our findings, hypoalbuminemia has been previously reported among SARS-CoV-2-infected children and adults, with an unrecognized mechanism (22).

Chest radiographic findings in symptomatic new-borns with COVID-19 infection have not been adequately described. In our study, radiological features were significant but not severe. Prominent hilar shadows with increased linear lung shadowing, mainly peri-hilar and pre-cordial, were detected in 34% of infants. Increased linear lung tracking with a tiny beading-like appearance, more peripherally distributed, and at the lower lobes, occurred in 28% of patients. Superadded GGO, scattered in two or more lobes of both lungs, occurred in 15.6% of patients. Rousan et al. (23) reported that the most common finding of chest radiographs in symptomatic children with COVID-19 infection was peripheral ground-glass appearance.

A total 26/27 of our SARS-CoV-2-positive infants did not require admission to the intensive care unit, denoting mild disease. Only one SARS-CoV-2-positive infant with congenital heart disease with a large ventricular septal defect had congestive heart failure and developed severe respiratory distress that required PICU admission and assisted ventilation. On the other hand, 18% of SARS-CoV-2-negative infants required PICU admission and assisted ventilation.

One SARS-CoV-2-positive infant (1/41) had coinfection with *Klebsiella pneumoniae* (meningitis). Bacterial coinfection was previously described in SARS-CoV-2-positive infants. Mclaren et al. described two SARS-CoV-2-positive infants with *Escherichia coli* urinary tract infections (UTIs) (24).

In a previous study of 74 SARS-CoV-2-positive children, coinfection with other common respiratory viral pathogens occurred in 34 children (51%) tested for common respiratory pathogens (25). Four of our SARS-CoV-2-positive infants were tested, and none of them had viral coinfection.

The decision to administer antibiotic therapy was based on daily clinical assessment and laboratory data of the infants. SARS-CoV-2-positive infants required a shorter duration of antibiotic therapy compared to SARS-CoV-2-negative infants. These findings may be explained by the more severe symptoms, positive bacterial cultures (UTI), and other laboratory findings (high ANC and CRP) in the SARS-CoV-2-negative group than in the SARS-CoV-2-positive infants (26).

In a systemic review, Rao et al. concluded that lower CT values may be associated with poor outcomes and that CT values can be used for predicting the clinical course for patients with COVID-19 infection (27); however, our SARS-CoV-2-positive neonates and young infants had significantly low CT values (high infectivity) despite their mild clinical symptoms and signs.

This study had some limitations. As this was a retrospective study, the retrospective design can introduce bias, including inadequate identification of analysis variables. The study had a small sample size, and the study period was short, limiting our findings' precision and generalizability. We did not have information regarding maternal SARS-CoV-2 status at the time of delivery because widespread screening was not yet being performed at our institution. We cannot comment on the possibility of vertical transmission or infection through early post-natal contact. Finally, we were unable to complete comprehensive respiratory pathogen panel testing because of the longer time needed to analyze the full panel; thus, we cannot comment on the prevalence or impact of viral coinfections. Despite these limitations, this study provided valuable insight as the first study to focus on the clinical and biochemical characteristics of infants with COVID-19 infection who presented with SLS.

## Conclusion

In febrile new-borns and young infants, COVID-19 presentation appeared to be mild (cough, nasal congestion, and lower total WBC count), but may simulate sepsis due to other causes. However, the low CT value of these infants suggested high infectivity due to the high viral load. Screening new-borns with SLS for SARS-CoV-2 is recommended during the COVID-19 pandemic.

## Data Availability Statement

The original contributions presented in the study are included in the article/supplementary material, further inquiries can be directed to the corresponding author.

## Ethics Statement

The study was conducted in conformance with the principles of the Declaration of Helsinki, Good Clinical Practice (GCP) and within the laws and regulations of MOPH in Qatar after the approval of IRB committee in HGH, Doha, Qatar with approval number of MRC-01-20-566.

## Author Contributions

MH, AK, SM, and AS conceptualized and designed the study, drafted the initial manuscript, supervised data collection and analysis, and reviewed and revised the manuscript. OA, TA, and HA designed the data collection instruments, collected, and interpreted the data. EA, AA, and MA reviewed and revised the manuscript. All authors approved the final manuscript as submitted and agree to be accountable for all aspects of the work.

## Conflict of Interest

The authors declare that the research was conducted in the absence of any commercial or financial relationships that could be construed as a potential conflict of interest.

## Abbreviations

SLS: Sepsis-Like Syndrome
COVID-19: Coronavirus disease-2019
SARS-CoV-2: severe acute respiratory syndrome coronavirus 2
PCR: Polymerase chain reaction
LP: lumbar puncture
CSF: Cerebrospinal fluid
ANC: Absolute neutrophil count
CT: Cycle threshold
ALT: Alanine Aminotransferase
AST: Aspartate Aminotransferase
PICU: Pediatric Intensive Care Unit
GGO: Ground glass opacities
UTI: urinary tract infection.
